# Evaluation of the pharmacokinetic-pharmacodynamic relationship of praziquantel in the *Schistosoma mansoni* mouse model

**DOI:** 10.1371/journal.pntd.0005942

**Published:** 2017-09-21

**Authors:** Nada Abla, Jennifer Keiser, Mireille Vargas, Natalie Reimers, Helmut Haas, Thomas Spangenberg

**Affiliations:** 1 Merck Global Health Institute, Ares Trading S.A., a subsidiary of Merck KGaA (Darmstadt, Germany), Coinsins, Switzerland; 2 Medicines for Malaria Venture, Geneva, Switzerland; 3 Department of Medical Parasitology and Infection Biology, Swiss Tropical and Public Health Institute, Basel, Switzerland; 4 University of Basel, Basel, Switzerland; 5 *helmin*Guard, Research Center Borstel, Borstel, Germany; University of Pennsylvania, UNITED STATES

## Abstract

After more than 40 years of use, Praziquantel (PZQ) still remains the drug of choice for the treatment of intestinal and urogenital schistosomiasis. Its anti-parasitic activity resides primarily in the (*R*)-enantiomer. Hitherto neither the molecular target nor the pharmacokinetic-pharmacodynamic relationship have been fully elucidated. Here we investigated the efficacy and pharmacokinetics of PZQ in the *Schistosoma mansoni* mouse model to determine the key factors that drive its efficacy. Dose-response studies with racemic PZQ with or without addition of an irreversible pan-cytochrome P450 (CYP) inhibitor, 1-aminobenzotriazole (ABT), were performed. In addition, efficacy of PZQ in the presence of the CYP inducer, dexamethasone (DEX), was determined. Plasma samples were obtained by tail vein bleeding at 4 time points. The (*R*)-PZQ levels were determined using a LC-MS/MS method. Non-compartmental pharmacokinetic analysis was performed using PKsolver. In addition, experiments using an enhanced *in vitro* assay were conducted. We found that the use of ABT increased (*R*)-PZQ plasma exposures in the systemic circulation by ~10 to 20 fold but the latter were not predictive of efficacy. The use of DEX decreased plasma exposures of (*R*)-PZQ in the systemic circulation by ~10 fold without reducing efficacy. We extrapolated the (*R*)-PZQ concentrations in mouse portal vein / mesenteric veins from the systemic exposures and found that a free exposure of (*R*)-PZQ of ~ 20 μM*h in the portal vein was needed to obtain a worm burden reduction >60%. It is suggested that the high (*R*)-PZQ concentrations available before the hepatic first pass metabolism drive the efficacy against *S*. *mansoni* adult worms residing in the mesenteric veins. It is then possible that the current dosing regimen of 40 mg/kg in preventive chemotherapy programs may provide suboptimal concentrations in low-weight patients such as children, due to smaller total amounts of drug administered, and may consequently result in lower cure rates.

## Author summary

After more than 40 years of use, Praziquantel (PZQ) still remains the drug of choice for the treatment of intestinal and urogenital schistosomiasis. However the relationship between pharmacokinetics and efficacy is not elucidated. For adults infected with intestinal schistosomiasis, a single oral dose of PZQ at 40 mg/kg is generally recommended by the World Health Organisation for preventive chemotherapy programs. By reducing hepatic metabolism with a pan-CYP inhibitor, we studied the systemic plasma exposure relationship versus activity in the *Schistosoma mansoni* mouse model. For *S*. *mansoni* infections where the worms are located in the mesenteric veins, we found that systemic plasma exposure was not predictive of efficacy, whereas estimated portal vein concentration of PZQ acts as a good predictor for the efficacy. This hypothesis was confirmed by pre-treatment of mice with a CYP inducer which led to a ~10 fold decrease in systemic plasma exposure while maintaining the efficacy of PZQ. This implies that the critical parameters are the rate and amount of PZQ absorbed, in order to achieve the required high local concentrations in the mesenteric veins. Therefore in pediatric patients the current dosing regimen of 40 mg/kg may provide suboptimal concentrations and consequently result in lower cure rates in *S*. *mansoni* infections.

## Introduction

Schistosomiasis [1–3] causes significant morbidity and mortality in subtropical and tropical regions of the world and estimates show that at least 258 million people required preventive treatment for schistosomiasis in 2014 [4]. Today treatment and control relies essentially on Praziquantel and gaps in the access plan to pre-school aged children remain an important issue along with an adequate pediatric formulation [5].

PZQ (1975) is administered as a racemate with the (*R*)-enantiomer (also described as L-PZQ) primarily accounting for the anti-parasitic activity [6]. PZQ has shown activity against all *Schistosoma* species but on adult worms only [7,8]. However after more than 40 years of use, neither the molecular target nor the pharmacokinetic-pharmacodynamic (PK/PD) relationship have been elucidated [9,10]. These lacking data hamper progression of much needed new chemical entities and may also raise questions regarding the suboptimal response of PZQ observed in some published pediatric studies [11,12]. To investigate the efficacy and pharmacokinetics of PZQ in a preclinical setting, the well-established *Schistosoma mansoni* mouse model is probably the most suited as it uses the most predominant species infecting humans with intestinal schistosomiasis and resides also in the same target organ, *i*.*e*. the mesenteric veins [13]. Besides obvious anatomical differences, two notable discrepancies are of course the immunity and the clearance which is greater in mice, although metabolic pathways are similar, mediated by cytochrome P450 (CYP) enzymes [14]. To circumvent rapid phase I metabolism, mice can be treated with 1-aminobenzotriazole (ABT), an irreversible pan-CYP inhibitor, with the intent to increase the systemic plasma exposure and enable to draw correlations between systemic exposure and efficacy [15]. Then additional insights may be obtained by the route of administration of ABT: an oral (*p*.*o*.) administration of ABT will reduce the intestinal and hepatic CYP-mediated metabolism whereas intravenous (*i*.*v*.) administration of ABT should predominantly reduce liver metabolism. After *p*.*o*. administration and before hepatic first pass, PZQ will transit through the mesenteric veins where most of the adult *S*. *mansoni* worms are located. To enhance the understanding of the PK/PD relationship, an opposite strategy can be applied, by pre-treating the mice with dexamethasone (DEX), an inducer of several CYPs, before PZQ administration to try to decrease systemic plasma exposures. In this study, results of the above mentioned *in vivo* strategies are disclosed and supported by *in vitro* parasitological works.

## Methods

### In vivo study design and procedure

#### Ethics considerations

*In vivo* studies were conducted at the Swiss TPH, Basel, and approved by the veterinary authorities of the Canton Basel-Stadt (permit no. 2070) based on Swiss cantonal (Verordnung Veterinäramt Basel-Stadt) and national regulations (the Swiss animal protection law (Tierschutzgesetz).

Blood sampling of healthy subjects for *in vitro* experiments conducted at *helmin*Guard was made upon informed written consent.

#### *In vivo* studies

Three-week old NMRI mice were purchased from Charles River (Sulzfeld, Germany) and allowed to adapt under controlled conditions (temperature ca. 22°C; humidity ca. 50%; 12-hour light and dark cycle; free access to rodent diet and water) for one week. Thereafter, they were infected subcutaneously with approximately 100 *S*. *mansoni* cercariae (Liberian strain), harvested from infected intermediate host snails *Biomphalaria glabrata* by exposure to light for 3 hours. Seven weeks post-infection, 6 mice were assigned to each PZQ treatment group, while 8 mice were left untreated to serve as controls.

Commercially available PZQ, *i*.*e*. racemic PZQ, (Sigma-Aldrich) was administered to the mice. For CYP inhibition studies, mice were pre-treated with *i*.*v*. or *p*.*o*. ABT (Sigma-Aldrich) 1 and 2 hours before *p*.*o*. administration of PZQ, respectively [15]. For CYP induction studies, mice were pre-treated with DEX (Acros Organics) for 3 days, with once a day *i*.*p*. administration of 100 mg/kg prior to *p*.*o*. administration of PZQ [16]. From each treated mouse 4 blood samples of 35 μL were collected from the tail vein at 0.5, 1.5, 2.5, 4 h post-dosing (0.5, 2.5, 4 and 7 h for groups treated with ABT). Five to 15 minutes after blood collection, samples were centrifuged at 2500 g for 5 minutes at room temperature. Blood cells were discarded and the obtained plasma (approximately 10–20 μL) was deep frozen and stored at –20°C. Three weeks post treatment mice were sacrificed by CO_2_ asphyxiation and dissected. The mesenteric tissue and the liver were collected. Adult worms were removed from the hepatic portal system and the mesenteric veins. The liver was flattened and examined for the presence of worms. Worms in the liver and mesenteric veins were recorded. The worms were washed, sexed, and counted using a binocular microscope. The worm burden (WB) of treated animals was calculated and compared with the worm burden of control mice (n = 8), which have been infected simultaneously but have not been treated. The worm burden reduction (WBR) is calculated as follows: WBR [%] = 100%—(100% / WB_control_ * WB_treatment_). The vehicle for *p*.*o*. application was 7% Tween / 3% ethanol, for *i*.*v*. application 2% DMSO / 20% Hydroxypropyl-beta-cyclodextrin and for *i*.*p*. administration was corn oil. Control mice were not treated.

### Pharmacokinetic analyses

(*R*)-PZQ and (*S*)-PZQ levels were determined by LC-MS/MS at Merck KGaA, Darmstadt, Germany using an enantioselective method (HPLC column: Lux 3μ Cellulose-2, 150 x 2 mm, Phenomenex. Solvents were 80% 20 mM ammonium hydrogen carbonate and 20% acetonitrile + 0.1% diethylamine. Flow: 200 μL/min. Retention times for (*R*)-Praziquantel is 6.30 min and (*S*)-Praziquantel is 8.24 min). Non-compartmental pharmacokinetic analysis was performed using *PKsolver 2*.*0* [17].

For PZQ dose response studies, the fraction escaping hepatic metabolism, F_H,_ was estimated to be ~ 0.05 therefore we calculated the portal vein exposure as follows: Portal vein AUC_0-t_ = Systemic plasma AUC_0-t_ / F_H_

For CYP inhibition studies, we followed the hypothesis that ABT completely inhibited hepatic CYP-mediated metabolism therefore the portal vein exposure was: Portal vein AUC_0-t_ = Systemic plasma AUC_0-t_

### *In vitro* study design and procedure

#### *In vitro* culture of *S*. *mansoni*

*S*. *mansoni cercariae* (Liberian strain, University of Giessen) were mechanically transformed using routine procedures. *Schistosomula* were cultured following the protocol of Basch [18] (slightly modified) in Iscove’s modified Dulbecco’s medium (PAA Laboratories GmbH) containing 5 μg/mL insulin (Gibco), 50 μg/mL transferrin (Sigma Aldrich), 100 U/mL penicillin (PAA), and 100 μg/mL streptomycin (PAA) with 10% foetal calf serum, FCS (PAA). After 24 h, human peripheral blood mononuclear cells (PBMC) from healthy donors were added at a concentration of 8 × 10^2^ cells per μL. After 48 h, human serum (final concentration 20%) and whole blood (0.3%) were added to the culture system. Culture was performed at humidified air at 37°C and 6% CO_2_. *Schistosomula* developed into adult stage including pairing after about 5–6 weeks.

#### Drug testing

For assessment of efficacy, DMSO solutions of (*R*)-PZQ, (*S*)-PZQ, PZQ metabolites [6], ABT and DEX were added to adult worms at the concentrations indicated. Testing was performed in 24-well plates (10–15 worms per well, duplicates). Following defined time intervals, drugs added at various concentrations were largely removed from the culture by 6 to 8 x replacing 50% of the medium by means of an automated microplate washer (BioTek MultiFlo FX). Thereafter, 50% of medium/blood cells were replaced every 2–3 days. Worms cultured in 0.1% DMSO served as negative control. Racemic PZQ and mefloquine (SelleckChem) were used as positive controls. Evaluations were performed at 1.5 h, 24 h, and day 7.

#### Protein binding assay

PZQ protein binding was determined by Rapid Equilibrium Dialysis [19]. The experimental fraction unbound (F_ub_) for (*R*)-PZQ in mouse plasma was ~20% and for the *in vitro* assay ~50%. F_ub_ was used to calculate the free area under the curve: ^free^AUC = AUC * F_ub_.

#### Statistical method

Statistical comparisons were done using the Kruskal Wallis Test using Statsdirect statistical software at a significance level of p < 0.05.

## Results

### *In vivo* pharmacokinetic and efficacy findings

WBRs and key pharmacokinetic exposure parameters are presented in Table 1. Without ABT treatment, the 200 mg/kg PZQ dose resulted in a significant (68%) WBR when compared to untreated control mice and the 400 mg/kg dose yielded a 97% WBR with a plasma C_max_ of (*R*)-PZQ of 6.3 μM and AUC_0-t_ of 11.4 μM*h [20]. With ABT treatment through the *i*.*v*. or the *p*.*o*. route, mice dosed with a single oral dose of PZQ at 50 mg/kg, 100 mg/kg and 200 mg/kg presented an increase in the C_max_ and AUC_0-t_ of (*R*)-PZQ. Oral administration of ABT provided a greater increase in exposure than *i*.*v*. administration for the 50 and 100 mg/kg PZQ doses. 50 mg/kg of PZQ *p*.*o*. with 50 mg/kg ABT *i*.*v*. achieved similar C_max_, and AUC_0-t_ to the positive control while the WBR was only 21%. Moreover, 100 mg/kg of PZQ *p*.*o*. with 20 mg/kg ABT *i*.*v*. showed ~2 times greater C_max_, and ~3 times greater AUC_0-t_ versus the positive control but only achieved a WBR of 60%. Finally treating the mice with ABT *i*.*v*. or *p*.*o*. before administering a 200 mg/kg dose of PZQ led to an improved WBR: 100% and 88%, after ABT *i*.*v*. and *p*.*o*., respectively, vs. 68% without ABT. However to achieve such efficacy a C_max_ increase by ~5 fold and an AUC_0-t_ increase by ~10 fold of the positive control was required.

**Table 1:** PZQ dose response studies with and without the CYP inhibitor, ABT.

PZQ dose (*p*.*o*., mg/kg)	ABT dose in mg/kg and route	(*R*)-PZQ C_max_, μM Median (range)	(*R*)-PZQ AUC_0-t_, μM*h Median (range)	Median worm burden reduction (%) (range)	Number of Mice included in analysis (n)^c)^	Statistical significance^e)^
**Negative control**^a)^	-	-	-	0%^a)^	8	N/A
**400; Positive control**	- ^b)^	**6.3** (3.6–21.0)	**11.4** (6.5–25.7)	**97** (84–100)	6	p<0.05
**200**	- ^b)^	**4.2** (0.5–9.9)	**7.1** (1.2–11.1)	**68** (65–71)	6	p<0.05
**200**	*20; i*.*v*.	**34.6** (33.3–48.0)	**113.5** (84.3–200.0)	**100** (97–100)	5	p<0.05
**200**	*50; p*.*o*.	**35.7** (20.5–38.7)	**127.6** (34.6–163.8)	**88** (54–100)	4	p<0.05
**100**	*20; i*.*v*.	**13.4** (6.9–33.9)	**31.2** (8.4–117.9)	**60** (12–73)	5	p<0.05
**100**	*50; p*.*o*.	**24.3** (11.5–40.3)	**53.2** (25.2–112.3)	**79** (54–95)	5	p<0.05
**50**	*50; i*.*v*.	**9.5** (8.2–10.8)	**14.2** (13.7–14.8)	**21** (14–28)	2^d)^	-
**50**	*50; p*.*o*.	**11.8** (10.3–19.9)	**22.3** (10.0–39.2)	**53** (0–71)	6	p<0.05

^a)^ Untreated NMRI mice inoculated with ~100 *S*. *mansoni* cercariae harbored a mean of 37.3 ± 8.8 worms with 95% of them located in the mesenteric veins;

^b)^ No ABT was administered;

^c)^ Several mice died from the severity of the infection;

^d)^ ABT *i*.*v*. at 50 mg/kg was not well tolerated and was replaced by 20 mg/kg in other groups.

^e)^ Statistical comparisons were done using the Kruskal Wallis Test at a significance level of p < 0.05

In a second study, a 400 mg/kg oral dose of PZQ provided a median WBR of 84%. When PZQ was given at the same dose to mice pre-treated with the CYP inducer DEX, a similar median WBR of 73% was obtained with a ~10 fold decrease in exposure of (*R*)-PZQ in comparison to the positive control (Table 2).

**Table 2:** PZQ efficacy in the presence of the CYP inducer, DEX.

PZQ dose (*p*.*o*., mg/kg)	DEX dose in mg/kg and route	(*R*)-PZQ C_max_, μM Median (range)	(*R*)-PZQ AUC_0-t_ μM*h Median (range)	Median worm burden reduction (%) (range)	Number of mice included in analysis (n)	Statistical significance^c)^
**Negative control**^a)^	-	-	-	0%^a)^	8	N/A
**400; Positive control**	- ^b)^	**11.7** (8.5–16.3)	**12·0** (9.8–17.2)	**84** (38–92)	4	p<0.05
**400**	*100; i*.*p*.	**1.2** (0.2–1.8)	**1·2** (0.2–1.7)	**73** (46–100)	4	p<0.05

^a)^ Untreated NMRI control mice inoculated with ~100 *S*. *mansoni* cercariae harbored a mean of 12.9 ± 6.3 worms with 95% of them located in the mesenteric veins;

^b)^ No DEX was administered.

^c)^ Statistical comparisons were done using the Kruskal Wallis Test at a significance level of p < 0.05

### Analysis of PK/PD relationship

**Fig 1:**
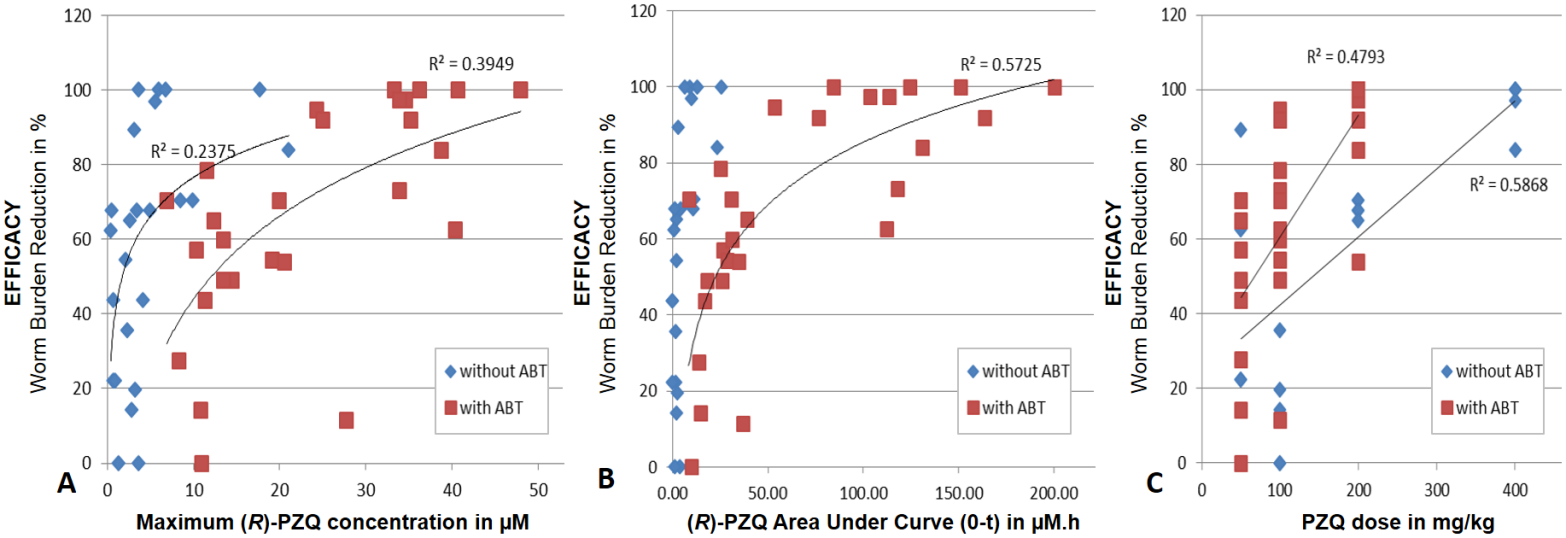
Correlation plots. Panel (A): %WBR vs. C_max_; Panel (B): %WBR vs. AUC_0-t_; Panel (C): %WBR vs. dose. Individual data points. In blue experiments without ABT; in red experiments with ABT.

Fig 1 depicts the plotting of individual mouse data for total plasma C_max_, AUC_0-t_ and dose as a function of WBR. In the absence of ABT, systemic plasma exposure (C_max_ and AUC_0-t_) does not correlate with the WBR but appears to correlate linearly with the dose. In the presence of ABT, a non-linear correlation could be observed between the AUC_0-t_ and the WBR.

In mice, estimation of the portal vein exposures in the groups not treated with ABT was made by assuming that 95% of PZQ was cleared after first pass in the liver (F_H_ = 0.05), and that the PK was dose linear. The assumption for F_H_ was based on the comparison of PK profiles between the 200 mg/kg dose with and without ABT *i*.*v*., where after *i*.*v*. administration of ABT the hepatic metabolism of PZQ was considered to be completely inhibited. For the groups treated with ABT, we assumed that *i*.*v*. and *p*.*o*. ABT completely inhibited hepatic CYP-mediated metabolism. Therefore the portal vein total exposure is equivalent to the systemic plasma exposure. As seen in Table 3, the values obtained from the groups not treated with ABT were in agreement with the corresponding experimental values using ABT *p*.*o*. and *i*.*v*.

**Table 3:** Estimation of (*R*)-PZQ portal vein exposures in dose response studies with and without the CYP inhibitor ABT.

PZQ dose (mg/kg)	(*R*)-PZQ AUC_0-t_ (μM*h), median (range) without ABT ^a)^	(*R*)-PZQ AUC_0-t_ (μM*h), median (range) with ABT *i*.*v*. ^b)^	(*R*)-PZQ AUC_0-t_ (μM*h), median (range) with ABT *p*.*o*. ^b)^
**50**	**27.5** (14.8–53.2)	**14.2** (13.6–14.8)	**22.3** (10.0–39.2)
**100**	**39.5** (24.9–76.8)	**31.2** (8.4–117.9)	**53.2** (25.1–112.3)
**200**	**142.1** (23.7–222.0)	**113.5** (84.3–200.0)	**127.6** (34.6–163.8)
**400**	**227.1** (129.0–513.1)	-	-

^a)^ Portal vein AUC_0-t_ = Systemic plasma AUC_0-t_ / F_H_; F_H_ is the fraction escaping hepatic metabolism and is estimated to be at 0.05.

^b)^ Assuming ABT completely inhibited hepatic CYP-mediated metabolism, Portal vein AUC_0-t_ = Systemic plasma AUC_0-t_

**Fig 2:**
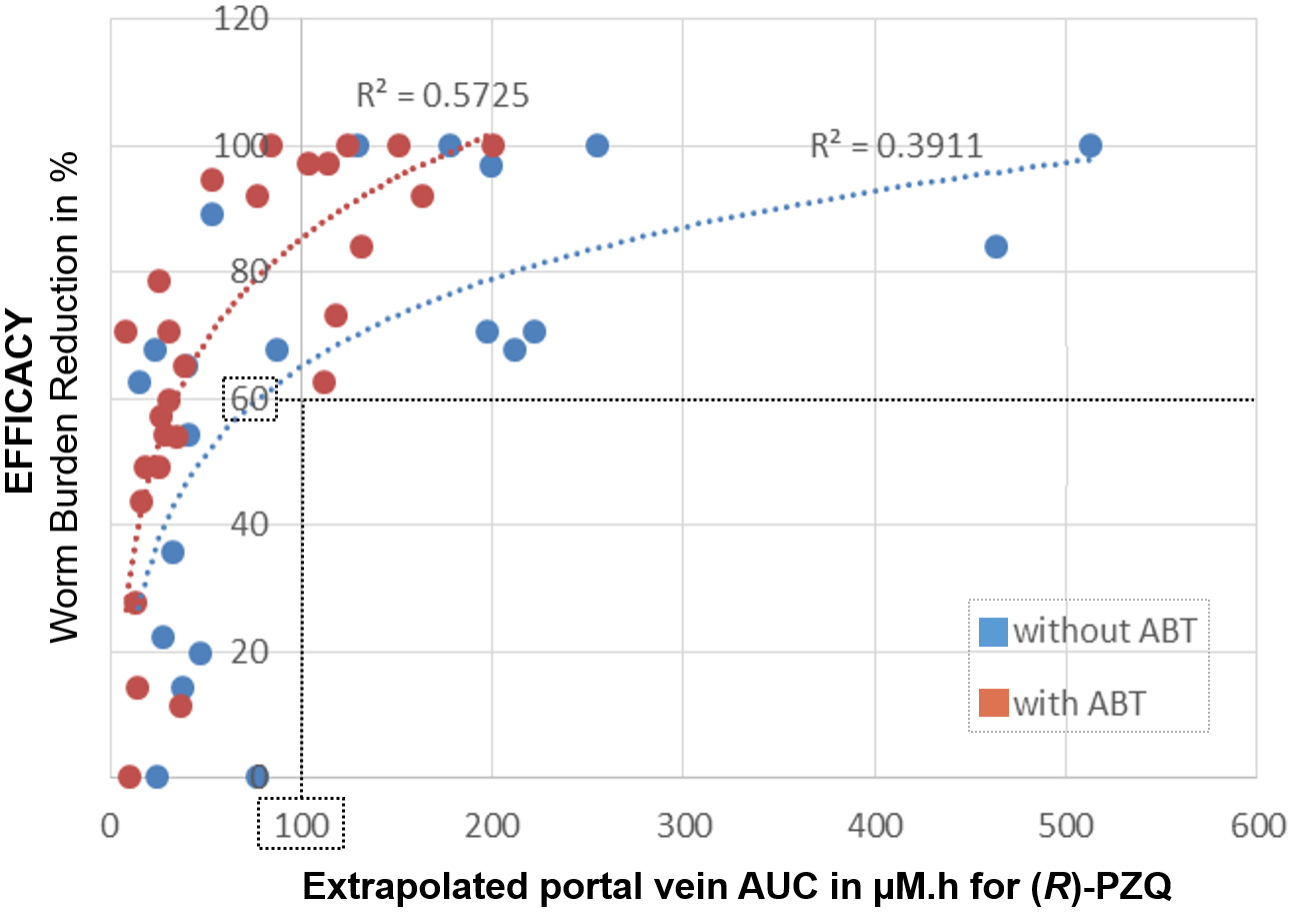
The correlation between efficacy expressed as worm burden reduction in % (y axis) and extrapolated AUC_0-t_ in the portal vein expressed in μM*h (x axis). Individual data points. In blue experiments without ABT; in red experiments with ABT.

The (*R*)-PZQ extrapolated portal vein exposures for treated mice were plotted against WBR. This correlation suggests that a portal vein AUC_0-t_ of (*R*)-PZQ greater than ~100 μM*h needs to be reached in order to achieve a WBR greater than 60%. (Fig 2). The corresponding free portal vein exposure (^free^AUC_0-t_) responsible for the activity was found to be ~20 μM*h as (*R*)-PZQ is ~20% unbound in mouse plasma.

### *In vitro* studies

Table 4 displays the activity of (*R*)-PZQ, (*S*)-PZQ, PZQ metabolites [6], ABT and DEX on adult *S*. *mansoni* in an enhanced *in vitro* assay.

**Table 4:** *In vitro* effects of (*R*)-PZQ, (S)-PZQ, PZQ metabolites [6], ABT and DEX against adult *S*. *mansoni*.

Compound	Conc	Incubation Time	SchistoTox^a)^^,^ ^b)^ at 1.5 h	SchistoTox at 24 h	SchistoTox at day 7
**(*R*)-PZQ**	2.1 μM	24 h	2	2	1.5
**(*S*)-PZQ**	2.1 μM	24 h	0	0	0
***Cis*-4-hydroxy-(*R*)-PZQ**	50.0 μM	24 h	ND	1.5	0.7
***Trans*-4-hydroxy-(*R*)-PZQ**	50.0 μM	24 h	ND	1.5	0
***Cis*-4-hydroxy-(*S*)-PZQ**	50.0 μM	24 h	ND	0	0
***Trans*-4-hydroxy-(*S*)-PZQ**	50.0 μM	24 h	ND	0	0
**ABT**	10 μM	24 h	0	0	0
**Dexamethasone**	10 μM	24 h	0	0	0
**DMSO**	0.1%	24 h	0	0	0
**PZQ**	10 μM	24 h	2	2	2
**Mefloquine**	10 μM	24 h	0	1.9	2

^a)^ SchistoTox scale indicates the degree of damage of adult *S*. *mansoni* worms at day 7 post exposure from 0 (healthy worms) to 2 (severe degeneration, death or no motility of the worms);

^b)^ 10–15 adult *S*. *mansoni* per well (24 well plate, n = 2)

Positive controls were PZQ and mefloquine, which, incubated at 10 μM for 24 h, resulted in severe degeneration, death or no motility of the worms whereas DSMO had no effect on worms. At 2.1 μM for 24 h (AUC = 50 μM*h; ^free^AUC = 25 μM*h; F_ub_ ~50%), (*R*)-PZQ led to shrinkage and paralysis of the worms with persisting damages at day 7. At a similar concentration of (*S*)-PZQ the worms became only transiently hyperactive.

When incubating all major previously disclosed metabolites formed, *i*.*e*. *cis*- and *trans* 4-hydroxy-PZQ of each enantiomer, at the very high concentration of 50 μM for 24 h (AUC = 1200 μM*h), only the (*R*)-PZQ derived metabolites revealed significant short term effects although worms could almost fully recover from it at day 7.

Finally, incubating ABT or DEX at 10 μM for 24 h had no effect on adult *S*. *mansoni*.

## Discussion

In the *S*. *mansoni* mouse model our study showed that a single dose of PZQ at 400 mg/kg was required to achieve excellent efficacy *i*.*e*. a WBR greater than 85% as compared to the control group in good agreement with previous work [21]. The very high metabolism of PZQ in the mouse [22] hampers achieving good systemic exposures with lower single, oral doses and we therefore hypothesized that pre-administration of a non-specific irreversible inhibitor of CYP such as ABT could reduce the clearance in mice and therefore increase PZQ concentrations and exposures (*i*.*e*. C_max_, AUC) in the plasma. The aim was to determine whether efficacy at doses lower than 400 mg/kg could be increased by increasing the plasma exposures for the same doses of PZQ. As expected, plasma exposures in the systemic circulation of the active enantiomer (*R*)-PZQ were increased by ~10 to ~20 fold when ABT was pre-administered. However, it appeared that ABT had limited impact on the efficacy: 50 mg/kg PZQ in mice pre-treated with ABT allowed reaching similar plasma exposures of (*R*)-PZQ to mice dosed with 400 mg/kg without any ABT pre-treatment, but the efficacy was lower (Table 1). The higher WBRs observed when ABT was administered *p*.*o*. over *i*.*v*. for 50 and 100 mg/kg PZQ doses suggest a role of intestinal metabolism, which is inhibited by oral ABT administration. ABT had limited impact on the t_1/2_ which ranged between 0.4–3 h after *p*.*o*. administration (Data available on request), in the presence and absence of ABT; however accurate assessment of the terminal phase was not possible due to the limited number of time points following the C_max_. Our data suggest that the systemic plasma exposure of PZQ is neither predictive nor the main contributor to the efficacy in the *S*. *mansoni* mouse model. This was strikingly demonstrated when administering the CYP inducer DEX prior to treatment with 400 mg/kg PZQ (Table 2). A similar level of efficacy was achieved with the same dose of PZQ, as compared to the positive control, while plasma exposures were decreased by ~10 fold.

It is therefore likely that high local concentrations of (*R*)-PZQ in the mesenteric veins, the target organ for adult *S*. *mansoni*, are required to achieve good efficacy, and that systemic exposures do not play a key role in the efficacy. The latter has been suggested by Xiao *et al*. in rabbits infected with *S*. *japonicum*; however, in this study, no correlation was observed with portal vein concentrations either [23].

Extrapolation of exposures in the portal vein, and thus in mesenteric veins, in mice led to the conclusion that ~100 μM*h (^free^AUC ~20 μM*h) was needed to reach significant worm burden reduction *i*.*e*. greater than 60%. Additional *in vitro* experiments showed that a ^free^AUC of *(R*)-PZQ of 25 μM*h produced long lasting damages to the worms, whereas the opposite enantiomer, (*S*)-PZQ, had only transient effects. Additionally, we have shown that 4-hydroxy-(*R*)-PZQ metabolites, formed at high concentration during first pass metabolism, affected only the worm viability on short term but are unlikely to be the main drivers for the efficacy. This is in agreement with previously published data [6]. An obvious caveat is that the *in vitro* assay cannot fully mimic the immune system of a living organism, a main contributor of the efficacy of PZQ [1, 2].

Regarding the relevance of these data in humans, like with any pharmacological model performed in preclinical species, the translation of mouse data to the human situation has to be done with caution. For example, the clearance is greater in the mouse and the contribution of the immune response may differ between species. However, the location of the parasite is the same in the mouse model and in human patients, and hepatic metabolism pathways are similar between both species [14]. Therefore, we believe that the major conclusion from this study, *i*.*e*. concentrations in the mesenteric/portal vein before first-pass hepatic metabolism are driving the efficacy in mice, could also be applicable to humans. As a consequence, we propose that PZQ efficacy in *S*. *mansoni* infections might be guided by the total administered PZQ dose. PZQ is highly permeable but has limited solubility therefore undergoes slow absorption from the gut lumen (BCS class II) [24]. Assuming similar absorption of PZQ is reached across age groups, low weight patients could then present a risk of reaching lower absolute concentrations of PZQ in the target veins and may provide an explanation for the observed reduced efficacy in some recent pediatric clinical trials [11,12].
